# Occluded Person Re-Identification via Multi-Branch Interaction

**DOI:** 10.3390/s25216526

**Published:** 2025-10-23

**Authors:** Yin Huang, Jieyu Ding

**Affiliations:** College of Computer Science and Technology, Qingdao University, Qingdao 266071, China; 2021010033@qdu.edu.cn

**Keywords:** occluded person re-identification, multi-head attention, multi-view information, mutual distillation

## Abstract

Person re-identification (re-ID) aims to retrieve images of a given individual from different camera views. Obstacles obstructing parts of a pedestrian’s body often result in incomplete identity information, impairing recognition performance. To address the occlusion problem, a method called Multi-Branch Interaction Network (MBIN) is proposed, which exploits the information interaction between different branches to effectively characterize occluded pedestrians for person re-ID. The method consists primarily of a hard branch, a soft branch, and a view branch. The hard branch enhances feature robustness via a unified horizontal partitioning strategy. The soft branch improves the high-level feature representation via multi-head attention. The view branch fuses multi-view feature maps to form a comprehensive representation via a dual-classifier fusion mechanism. Moreover, a mutual knowledge distillation strategy is employed to promote knowledge exchange among the three branches. Extensive experiments are conducted on widely used person re-ID datasets to validate the effectiveness of our method.

## 1. Introduction

Person re-identification (re-ID) is a technique for matching images of the same pedestrian from multiple non-overlapping camera views, being regarded as a specialized form of image retrieval [1,2]. As a key topic in computer vision, re-ID has attracted considerable interest from academia and industry, with diverse applications such as criminal investigations, rescue missions, and behavioral analysis [3,4,5,6,7,8]. Re-ID helps compensate for blind spots in multi-camera surveillance and mitigates the limitations of fixed equipment. However, pedestrians with different identities may appear visually similar, while those with the same identity may exhibit significant variations in appearance. Therefore, re-ID is a challenging task.

According to the strategy of feature extraction, re-ID methods can generally be divided into two categories: hand-crafted feature-based methods and deep learning-based methods. The former primarily focus on manually designed low-level visual features to represent identity information, with similarity measured using distance functions [9,10,11,12]. These methods can perform well but lack robustness to variations in illumination, occlusion, and pose, making them often unreliable for complex real-world scenarios. Deep learning-based methods mainly focus on learning high-level semantic feature representations automatically, including feature-based methods and metric-based methods. Feature-based methods train the network to perform a multi-class classification task, using the features from the last layer to represent the pedestrian [13,14,15,16,17,18]. Metric-based methods rely on loss functions that shape the distribution of feature distances between samples, encouraging the model to learn discriminative embeddings [19,20,21,22,23].

Occlusion is common in intelligent surveillance and traffic scenarios. Pedestrians are often partially obscured by other individuals or environmental objects such as walls, vehicles, trees, or billboards, especially in dynamic scenes. These occlusions reduce the visible regions of pedestrians, hindering the extraction of identity cues and increasing the difficulty of recognition. To address the occlusion issue in person re-ID, this study proposes a method named Multi-Branch Interaction Network (MBIN), which exploits the information interaction between different branches to effectively characterize occluded pedestrians. The proposed method mainly consists of three branches: a hard branch, a soft branch, and a view branch. The hard branch extracts local information via a partitioning strategy. The soft branch employs attention mechanisms to integrate multi-scale information. The view branch leverages multi-view information to form a comprehensive representation. Although the three branches extract features in different ways, the identity information remains highly consistent across branches. Therefore, a mutual distillation strategy is used to facilitate knowledge exchange among them.

The main contributions of this work are summarized as follows:

1. Multi-head attention is employed to supplement the high-level feature with discriminative cues.
2. A dual-classifier fusion mechanism is designed to adaptively assign weights to different views, generating a comprehensive pedestrian representation.
3. Mutual distillation is introduced to establish collaborative learning pathways across branches, enhancing the consistency of multi-branch features.
4. Extensive experiments are conducted on five public person re-ID datasets to demonstrate the effectiveness of the proposed method.

The rest of this paper is structured as follows: Section 2 introduces the related work; Section 3 presents the proposed method; Section 4 provides an experimental comparison and analysis; and Section 5 concludes this paper.

## 2. Related Works

### 2.1. Occluded Person Re-ID

Occluded person re-ID aims to retrieve pedestrians who are partially occluded in a query image across multiple non-overlapping camera views. According to the strategy of utilizing external visual cues, occluded person re-ID methods can generally be divided into two categories: external model-assisted methods and external model-free methods.

External model-assisted methods employ auxiliary tools to provide structured cues or region-level guidance [24,25,26,27]. Miao et al. [24] employed pose information to generate attention maps that help the model suppress occluded regions during both training and matching. Gao et al. [25] proposed a pose-guided attention mechanism to mine visible region information of pedestrians. Wang et al. [26] employed a keypoint estimation model for semantic part feature extraction and introduced adaptive-directional graph convolution to enhance the propagation of local semantic cues. Ma et al. [27] designed a framework that incorporates striped slices, patch grids, and pose-keypoint regions as local descriptors, using a Transformer to model contextual dependencies.

External model-free methods learn pedestrian representations by designing robust feature extraction mechanisms that do not rely on auxiliary tools [16,28,29,30,31,32]. Chen et al. [29] modeled the relationship between occlusion locations and occluded areas and designed an occlusion augmentation strategy to generate diverse occluded samples. Li et al. [14] adopted a Transformer-based architecture to capture pixel-wise correlations in feature maps and extract robust representations. Jia et al. [21] introduced a contrastive feature learning scheme to decouple occlusion-related and identity-related features. Huang et al. [17] inferred visible body parts using local–global semantic consistency and compensated for missing parts. Wang et al. [16] evaluated the quality of local features and generated global representations from unoccluded regions. Dong et al. [31] proposed an information propagation mechanism to transfer multi-view knowledge into single-view images. Tan et al. [32] partitioned training samples into identity and occlusion sets, then recombined them using an occlusion-aware intersection over union algorithm to synthesize realistic occluded images.

### 2.2. Distillation Learning

Distillation learning is a technique for model compression and knowledge transfer, aiming to improve the performance of a student model by transferring knowledge from a teacher model [33,34,35]. Hinton et al. [36] introduced the concept of knowledge distillation in 2015, using the outputs of large or ensemble models as soft targets for training smaller models and applying a temperature coefficient to smooth the probability distribution. Zhang et al. [37] proposed a deep mutual learning strategy, where multiple networks are trained jointly, using Kullback–Leibler (KL) divergence as a regularizer to enable bidirectional knowledge exchange. Zheng et al. [38] applied knowledge distillation to regularize the main network branch, allowing the model to eliminate its dependency on pose information at inference time, thus reducing the overall complexity of the re-ID framework. Sun et al. [39] introduced patch logit and patch relation distillation techniques to preserve patch-level semantics and inter-patch relationships, thereby mitigating catastrophic forgetting in lifelong re-ID. Zhu et al. [22] applied distillation to multi-view pseudo labels to enhance both global and local feature representations, alleviating supervision bias introduced by clustering in unsupervised re-ID.

## 3. The Proposed Method

**Figure 1:**
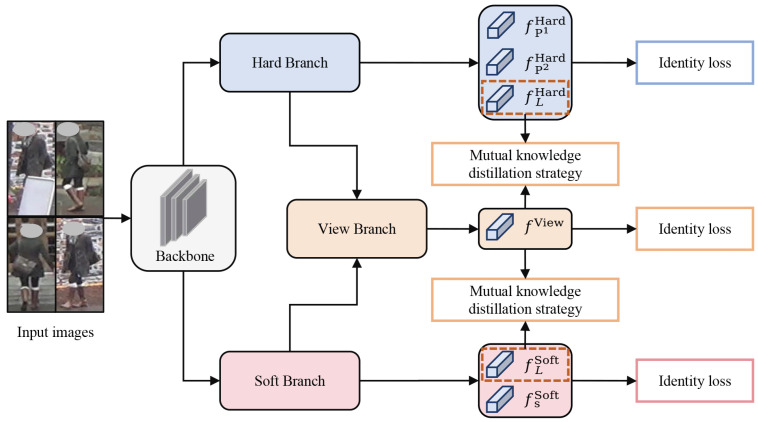
Overview of the proposed method. It comprises a backbone and three branches: the hard branch, the soft branch, and the view branch.

This study proposes a method named MBIN for occluded person re-ID that exploits the information interaction between different branches to effectively characterize occluded pedestrians. MBIN consists of three main branches: a hard branch, a soft branch, and a view branch. The hard branch employs a horizontal partitioning strategy to extract the local feature from different pedestrian regions. The soft branch employs multi-head attention to extract the cross-scale semantic feature. The view branch fuses local and cross-scale semantic features, and employs a dual-classifier fusion mechanism to integrate multi-view information. Additionally, a mutual knowledge distillation (MKD) strategy facilitates bidirectional knowledge exchange among branches, enhancing both the consistency and complementarity of the features. MBIN adopts ResNet-50 [40] as the backbone for feature extraction. The hard and soft branches share parameters in the first three stages of the backbone, but employ separate parameters in the fourth stage to learn more distinct features. Each branch is supervised using identity loss to guide discriminative feature learning. During inference, the proposed method employs single-view features for similarity measurement. An overview of the MBIN is shown in Figure 1.

### 3.1. Hard Branch

Localized body regions not only facilitate the distinction of human body parts but also mitigate the impact of intra-class appearance variations, thus enhancing recognition robustness. The hard branch leverages human structural priors by applying a horizontal partitioning strategy that segments the feature map into multiple regions for local feature extraction.

In the hard branch, global feature map $F_{L}^{\mathrm{Hard}}\in R^{C_{L}\times H_{L}\times W_{L}}$ is extracted from from the final stage *L* of the backbone network, where $C_{L}$, $H_{L}$, $W_{L}$ denote the dimensions of channels, height, and width, respectively. $F_{L}^{\mathrm{Hard}}$ is uniformly partitioned along the height dimension into $N_{p}$ horizontal stripes, each corresponding to a specific body region. $N_{p}$ is set to 2, which divides the body into two regions: the upper and lower segments. This study extracts the local features $F_{p^{1}}^{\mathrm{Hard}}$ and $F_{p^{2}}^{\mathrm{Hard}}$ from the upper and lower parts, respectively.

To obtain compact vector representations, Generalized Mean (GeM) [41] pooling is performed on $F_{L}^{\mathrm{Hard}}$, $F_{p^{1}}^{\mathrm{Hard}}$, and $F_{p^{2}}^{\mathrm{Hard}}$, yielding the features $f_{L}^{\mathrm{Hard}}$, $f_{p^{1}}^{\mathrm{Hard}}$, and $f_{p^{2}}^{\mathrm{Hard}}$ in hard branch. GeM is a differentiable pooling method that introduces a learnable parameter, allowing a smooth interpolation between average and max pooling. This enables adaptive feature aggregation that balances spatial smoothing with enhanced activation. The GeM operation is as follows:

(1) $$x_{i}={\left(\frac{1}{|F_{i}|}\sum_{f\in F_{i}}f^{\alpha }\right)}^{\frac{1}{\alpha }},$$

where α is a learnable parameter, $F_{i}$ represents the set of activations in the *i*-th feature map, and $|F_{i}|$ represents the number of spatial elements.

### 3.2. Soft Branch

**Figure 2:**
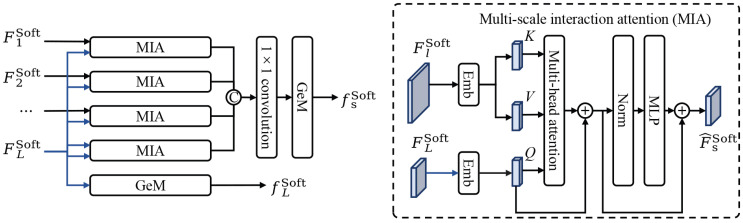
Illustration of the soft branch. MIA refers to the multi-scale interaction attention module. Emb refers to the embedding layer. Norm refers to layer normalization. © denotes the concatenation operation. ⊕ denotes the element-wise sum operation.

Convolutional neural networks provide hierarchical features, where shallow layers retain fine-grained textures and deep layers encode high-level semantic cues. Relying solely on single-scale features may overlook critical dependencies between local details and global context. Therefore, this study designs a Multi-scale Interaction Attention (MIA) module and adopts a cascaded fusion strategy to integrate multi-scale information. The soft branch consists primarily of multiple MIA modules and takes feature maps from different stages of the backbone network as input. The MIA module utilizes multi-head attention [42], establishing connections across different stages of the backbone, supplementing multi-granularity information for the high-level feature map. An illustration of the soft branch is shown in Figure 2.

The MIA module consists primarily of the embedding layer, the multi-head attention, and the Multi-Layer Perceptron (MLP). It takes the shallow feature map $F_{l}^{\mathrm{Soft}}$ and high-level feature map $F_{L}^{\mathrm{Soft}}$ as input, where l∈{1,2,…,L} denotes the index of the backbone stage.

In the embedding layer, bilinear interpolation is applied to downsample $F_{l}^{\mathrm{Soft}}$ to match the spatial resolution of $F_{L}^{\mathrm{Soft}}$. Both feature maps are projected into one-dimensional token sequences $T\in R^{U\times D}$ through a patch embedding operation, where *U* denotes the sequence length and *d* is the embedding dimension. The embedding process is as follows:

(2) $$T_{l}^{\mathrm{Soft}}=\mathrm{Norm}\left(\mathrm{PatchEmbedding}\left(F_{l}^{\mathrm{Soft}}\right)\right),$$

where Norm(·) represents layer normalization. $T_{L}^{\mathrm{Soft}}$ are projected as query vector *Q*, while $T_{l}^{\mathrm{Soft}}$ are projected as key vector *K* and value vector *V*, as follows:

(3) $$Q_{L,j}=T_{L}^{\mathrm{Soft}}W_{l,j}^{Q},\;K_{l,j}=T_{l}^{\mathrm{Soft}}W_{l,j}^{K},\;V_{l,j}=T_{l}^{\mathrm{Soft}}W_{l,j}^{V},$$

where $j\in \{1,2,\ldots ,N_{h}\}$ is the index of projection heads, and $W_{l,j}^{Q}$, $W_{l,j}^{K}$, and $W_{l,j}^{V}\in R^{D\times d}$ are learnable linear projection matrices.

This study adopts multi-head attention [43] to enable feature interaction across different granularities. Multi-head attention computes attention independently across multiple subspaces, allowing the model to capture diverse contextual patterns and long-range dependencies. The output of the *j*-th attention head at the *l*-th stage is computed as follows:

(4) $$head_{l,j}=\mathrm{Softmax}\left(\frac{Q_{L,j}K_{l,j}^{T}}{\sqrt{d_{k}}}\right)V_{l,j},$$

where $d_{k}$ is the dimensionality of *K*. The outputs of all attention heads are concatenated to form the attention output for *l*-th stage:

(5) $$MHA_{l}=\mathrm{Concat}\left(head_{l,1},head_{l,2},\ldots ,head_{l,N_{h}}\right).$$

After multi-head attention, layer normalization and residual connections are applied, followed by an MLP composed of two fully connected layers with a GELU activation, which enhances nonlinear modeling and representation capacity.

Subsequently, the one-dimensional sequence is reshaped back into the two-dimensional feature map. Feature maps from different stages are concatenated along the channel dimension and compressed using a 1×1 convolution to generate the cross-scale semantic feature map $F_{s}^{\mathrm{Soft}}$. Finally, GeM is applied to $F_{L}^{\mathrm{Soft}}$ and $F_{s}^{\mathrm{Soft}}$, yielding the features $f_{L}^{\mathrm{Soft}}$ and $f_{s}^{\mathrm{Soft}}$.

### 3.3. View Branch

Due to variations in camera viewpoints, pedestrian poses, and occlusions, some images may suffer from significant information loss, which hinders the extraction of discriminative features. Therefore, this study designs the View Integration (VI) module based on a dual-classifier fusion mechanism to evaluate and fuse information from multiple views. The VI module consists primarily of a hard-branch classifier and a soft-branch classifier, and takes features from two branches as input and outputs confidence scores. The images with higher confidence scores contribute more significantly to the aggregated representation, while those with lower confidence are down-weighted to mitigate the influence of less informative or occluded views.

The hard branch emphasizes structural cues of the human body, while the soft branch captures multi-scale details. The feature maps from these branches are complementary. Thus, $F^{\mathrm{Hard}}$ and $F^{\mathrm{Soft}}$ are concatenated along the channel dimension, followed by a 1×1 and a 3×3 convolution to generate the fused feature map ${\hat{F}}^{\mathrm{View}}$.

Images captured from viewpoints with more complete visibility of body regions typically contain more identity cues. Thus, the confidence score is estimated for each image. The feature maps from *M* different viewpoints of pedestrians with the same identity are denoted as $\left\{{\hat{F}}_{1}^{\mathrm{View}},{\hat{F}}_{2}^{\mathrm{View}},\ldots ,{\hat{F}}_{M}^{\mathrm{View}}\right\}$, where ${\hat{F}}_{m}^{\mathrm{View}}$ represents the feature map from the *m*-th viewpoint. Each of these feature maps is then fed into both the hard branch classifier and soft branch classifier, producing predicted probabilities $p_{m}^{\mathrm{Hard}}$ and $p_{m}^{\mathrm{Soft}}$, respectively. The confidence score $W_{m}^{\mathrm{View}}$ is computed as follows:

(6) $$W_{m}^{\mathrm{View}}=\frac{\exp(p_{m}^{\mathrm{Hard}}+p_{m}^{\mathrm{Soft}})}{\sum_{m=1}^{M}\exp\left(p_{m}^{\mathrm{Hard}}+p_{m}^{\mathrm{Soft}}\right)}.$$

Next, a weighted fusion of the feature maps is performed to generate a multi-view complementary feature map:

(7) $$F^{\mathrm{View}}=\sum_{m=1}^{M}W_{m}^{\mathrm{View}}{\hat{F}}_{m}^{\mathrm{View}}.$$

Finally, the GeM operation is applied to $F^{\mathrm{View}}$ to generate the feature $f^{\mathrm{View}}$.

### 3.4. Mutual Knowledge Distillation Strategy

In a multi-branch architecture, different branches typically extract complementary feature representations. To effectively integrate the strengths of each branch, this study designs an MKD strategy that enables knowledge transfer between branches.

The MKD strategy employs a bidirectional learning mechanism to collaboratively optimize branches’ feature representations. On the one hand, the view branch acts as a teacher model, providing comprehensive person representations to supervise both the hard and soft branches, alleviating the issue caused by the absence of multi-view images with the same identity during inference. Let $p^{\mathrm{Hard}}$, $p^{\mathrm{Soft}}$, and $p^{\mathrm{View}}$ denote the predicted probabilities corresponding to the features $f_{L}^{\mathrm{Hard}}$, $f_{L}^{\mathrm{Soft}}$, and $f^{\mathrm{View}}$, respectively. To effectively transfer the information, the view distillation loss $L_{\mathrm{MKD}}^{V}$ is formulated as follows:

(8) $$L_{\mathrm{MKD}}^{V}=\mathrm{KL}\left(p^{\mathrm{View}}\left|\right|p^{\mathrm{Hard}}\right)+\mathrm{KL}\left(p^{\mathrm{View}}\left|\right|p^{\mathrm{Soft}}\right),$$

where KL(·||·) represents the KL divergence, which quantifies the difference between probability distributions. The divergence is calculated as follows:

(9) $$\mathrm{KL}(p_{i}\left|\right|p_{j})=\sum_{m=1}^{N_{c}}p_{i}^{m}\log\frac{p_{i}^{m}}{p_{j}^{m}},$$

where $N_{c}$ denotes the number of identity classes. Knowledge is transferred from the view branch to the hard and soft branches through the view distillation loss, guiding them to learn multi-view information reasoning. During the testing phase, relationships between the target pedestrian and others are generally not directly available. Therefore, MBIN employs a single image as the query and extracts features from both the hard and soft branches to measure similarity.

On the other hand, the hard and soft branches serve as teacher models, offering discriminative and refined feature representations to enhance the view branch’s learning process. To effectively transfer the information, the refinement distillation loss $L_{\mathrm{MKD}}^{R}$ is formulated as follows:

(10) $$L_{\mathrm{MKD}}^{R}=\mathrm{KL}\left(p^{\mathrm{Hard}}\left|\right|p^{\mathrm{View}}\right)+\mathrm{KL}\left(p^{\mathrm{Soft}}\left|\right|p^{\mathrm{View}}\right).$$

The MKD loss is the sum of the above two components:

(11) $$L_{\mathrm{MKD}}=L_{\mathrm{MKD}}^{V}+L_{\mathrm{MKD}}^{R}.$$

### 3.5. Loss Function

During the training phase, a combination of identity loss and MKD loss is applied to optimize the network. The cross-entropy loss function is adopted to calculate the identity loss, minimizing the difference between the model’s predicted probability distribution and the ground truth. This function treats identity labels as supervisory signals, transforming the re-ID task into an image classification problem. To mitigate model overfitting, this study introduces label smoothing regularization [44], which smooths the pedestrian labels.

The cross-entropy loss is calculated as follows:

(12) $$L_{\mathrm{CE}}=-\sum_{i=1}^{N_{c}}q_{i}\log p_{i},$$

where $N_{c}$ denotes the number of identity classes, $p_{i}$ represents the model’s predicted probability for the *i*-th class, and $q_{i}$ represents the smoothed label for the *i*-th class, calculated as follows:

(13) $$q_{i}=\left\{\begin{array}{cc} 1-\epsilon +\frac{\epsilon }{N_{c}}, & y=i,\\ \frac{\epsilon }{N_{c}}, & y\neq i, \end{array}\right.$$

where *y* represents the true label of the sample, ϵ∈[0,1] is the smoothing parameter, which is set to 0.1. To learn discriminative pedestrian features, the cross-entropy loss is applied to three branches. The overall identity loss $L_{\mathrm{ID}}$ is the sum of the cross-entropy losses from each branch:

(14) $$L_{\mathrm{ID}}=L_{\mathrm{CE}}^{\mathrm{Hard}}+L_{\mathrm{CE}}^{\mathrm{Soft}}+L_{\mathrm{CE}}^{\mathrm{View}}.$$

During the training phase, the total loss is calculated as follows:

(15) $$L_{\mathrm{total}}=L_{\mathrm{ID}}+\lambda_{\mathrm{MKD}}L_{\mathrm{MKD}},$$

where $\lambda_{\mathrm{MKD}}$ is a hyperparameter for the balance loss.

## 4. Experimental Results and Analysis

### 4.1. Datasets and Evaluation Metrics

#### 4.1.1. Datasets

To validate the effectiveness of our proposed method, we conducted experiments on three occlusion re-ID datasets and two holistic re-ID datasets. The occlusion re-ID datasets include Occluded-DukeMTMC [24], Occluded-REID [24], and P-DukeMTMC-reID [45], while the holistic re-ID datasets include Market-1501 [46] and DukeMTMC-reID [47]. The details of the datasets are provided in Table 1.

Occluded-DukeMTMC is an occlusion dataset containing images captured from eight different camera views. It includes 15,618 training images for 702 identities. For testing, it contains 2210 query images for 519 identities and 17,661 gallery images for 1110 identities.

Occluded-REID is a small-scale occlusion dataset collected on campus using a mobile camera. It contains 2000 images of 200 identities, each with 5 full-body and 5 occluded images. Following occluded re-ID methods [24,48], 1000 images from 100 identities are randomly sampled for training, while the remaining images are used for testing. During testing, occluded images are used as queries, and full-body images are used as galleries. The experiment is repeated 10 times on the dataset, and the average results are reported.

P-DukeMTMC-reID is an occlusion dataset collected from eight camera viewpoints. It includes 12,927 training images for 665 identities. For testing, it contains 2163 query images for 634 identities and 9053 gallery images for 634 identities.

Market-1501 is a full-body dataset collected at Tsinghua University using images from five high-resolution cameras and one low-resolution camera. It consists of 12,936 training images for 751 identities. For testing, it contains 12,936 query images for 750 identities and 19,732 gallery images for 750 identities.

DukeMTMC-reID is a full-body dataset collected at Duke University from eight different camera viewpoints. It contains 16,522 training images for 702 identities. For testing, it contains 2228 query images of 702 identities and 17,661 gallery images of 1110 identities.

#### 4.1.2. Evaluation Metrics

This study adopts Mean Average Precision (mAP) and Cumulative Matching Characteristics (CMC) as evaluation metrics to assess the performance of the methods. The CMC protocol evaluates performance on the Rank-k, which represents the probability of retrieving the correct object within the top-k positions. The mAP reflects the ranking quality by considering the positions of all correct matches in the retrieval list.

### 4.2. Experimental Setup

All experiments are conducted on a single GTX 3060 GPU on the Ubuntu 16.04 operating system. The algorithm is implemented on Python (Version 3.9) using the PyTorch (Version 2.2) framework, and the integrated development environment used is Visual Studio Code (Version 1.105). MBIN takes about 6, 0.4, 5, 5, and 6 h for training on Occluded-DukeMTMC, Occluded-REID, P-DukeMTMC-reID, Market-1501, and DukeMTMC-reID, respectively. During inference, MBIN extracts features from each image in about 3 milliseconds.

During training, the height and width of the input image are scaled to 256 and 128 pixels, respectively, following previous occluded re-ID methods [17,21]. Image augmentation techniques, including random flipping, random erasing, and random padding, are applied to increase the robustness of the model. The image batch size is set to 64, and a balanced sampling strategy is used, where 8 pedestrian identities are randomly selected, with 8 images sampled per identity. During training, the number of viewpoints *M* is set to 4, and the random sampling strategy is used to select pedestrian images with the same label within each batch for multi-view information integration.

ResNet-50 [40] is employed as the backbone network, initialized with parameters pre-trained on ImageNet [49], and modified by reducing the stride of the last convolutional block to 1. The initial learning rate is set to 0.0003, and it is reduced by a factor of 0.1 at the 40th and 70th epochs, respectively. The Adam optimizer is used with momentum 0.9 and weight decay 0.0005. The number of epochs is set to 120. The seed is set to 3407 to ensure the reproducibility of the experiments. In the multi-head attention, the number of heads, embedding dimension, and projection size are set to 8, 768, and 64, respectively. The patch sizes used in PatchEmbedding for the four backbone stages are 4×4, 2×2, 1×1, and 1×1, respectively. The classifier consists of a batch normalization layer followed by a fully connected layer.

### 4.3. Performance Comparison

This study compares the proposed method with several related methods for both occlusion and holistic person re-ID tasks. The comparison results are presented in Table 2 and Table 3. In addition, we do not use post-processing techniques such as re-rank.

This study investigates the recognition performance of the proposed method on three occlusion datasets. The performance comparison results are presented in Table 2. On the Occluded-DukeMTMC dataset, MBIN achieves a mAP of 59.1% and Rank-1 of 71.2%. Compared with the DPEFormer [50], which dynamically selects human body part information free from occlusions at the patch token level, mAP and Rank-1 improve by 0.2% and 1.3%, respectively. Compared to MVIIP [31], mAP and Rank-1 improve by 1.8% and 2.6%, respectively. On the Occluded-REID dataset, MBIN achieves a mAP of 87.1% and Rank-1 of 92.8%. Compared to SCAT [51], which is based on the Transformer structure, MBIN improves both mAP and Rank-1. Compared to CAAO [4], mAP and Rank-1 improve by 3.7% and 5.7%, respectively. On the P-DukeMTMC-reID dataset, MBIN achieves a mAP of 82.5% and Rank-1 of 93.0%. Compared to FED [30], mAP and Rank-1 improve by 2% and 1.5%, respectively. These experimental results demonstrate that the proposed method significantly enhances the person re-ID performance in complex occluded environments.

This study investigates the recognition performance of the proposed method on two holistic datasets. The performance comparison results are presented in Table 3. On the Market-1501 dataset, MBIN achieves a mAP of 89.1% and Rank-1 of 96.1%. Compared to PAT [52], which mines local visual information, mAP and Rank-1 improve by 7.6% and 3.7%, respectively. Compared to ViT-SPT [32], mAP and Rank-1 improve by 2.9% and 1.6%, respectively. On the DukeMTMC-reID dataset, MBIN achieves a mAP of 80.1% and Rank-1 of 91.2%. Compared to PGFA [38], mAP and Rank-1 improve by 0.6% and 1.6%, respectively. These experimental results demonstrate that the proposed method not only improves recognition accuracy in the occlusion task but also performs well in the holistic person re-ID task. This further demonstrates the effectiveness of the proposed method.

**Table 2:** Performance comparison on the Occluded-DukeMTMC, Occluded-REID, and P-DukeMTMC-reID datasets.

Method	Occluded-DukeMTMC		Occluded-REID		P-DukeMTMC-reID	
	mAP	Rank-1	mAP	Rank-1	mAP	Rank-1
DSR [28]	30.4	40.8	62.8	72.8	68.0	73.7
PGFA [24]	37.3	51.4	-	-	72.4	85.7
PVPM [25]	37.7	47.0	61.2	70.4	-	-
HOReID [26]	43.8	55.1	70.2	80.3	-	-
Pirt [27]	50.9	60.0	-	-	-	-
PGFL-KD [38]	54.1	63.0	70.3	80.7	-	-
IGOAS [48]	49.4	60.1	81.1	91.6	-	-
OAMN [29]	46.1	62.6	-	-	77.4	86.0
DPD-PAT [14]	53.6	64.5	72.1	81.6	-	-
TransReID [43]	55.7	64.2	67.3	70.2	68.6	71.3
FED [30]	56.4	68.1	79.3	86.3	80.5	83.1
MHSANet [15]	44.8	59.7	-	-	37.6	67.9
QPM [16]	53.3	66.7	-	-	74.4	89.4
CAAO [4]	55.8	67.8	83.4	87.1	79.5	90.5
DRL-Net [21]	50.8	65.0	-	-	-	-
RTGAT [17]	50.1	61.0	51.0	71.8	74.3	85.6
SCAT [51]	54.9	62.8	76.1	80.4	-	-
ViT-SPT [32]	57.4	68.6	81.3	86.8	-	-
MVIIP [31]	57.3	68.6	77.4	85.5	79.0	91.5
DPEFormer [50]	58.9	69.9	79.5	87.0	-	-
MBIN (Ours)	59.1	71.2	87.1	92.8	82.5	93.0

‘-’ denotes that no reported result is available.

**Table 3:** Performance comparison on the Market-1501 and DukeMTMC-reID datasets.

Method	Market-1501		DukeMTMC-reID	
	mAP	Rank-1	mAP	Rank-1
BOT [19]	85.7	94.1	76.4	86.4
PGFA [24]	76.8	91.2	79.5	89.6
HOReID [26]	84.9	94.2	75.6	86.9
ISP [53]	88.6	94.9	78.4	88.9
Pirt [27]	86.3	94.1	77.6	88.9
PGFL-KD [38]	87.2	95.3	79.5	89.6
DPD-PAT [14]	88.0	95.4	78.2	88.8
CAAO [4]	87.3	95.1	77.5	88.9
RTGAT [17]	88.2	93.3	76.9	88.0
DRL-Net [21]	86.9	94.7	76.6	88.1
PAT [52]	81.5	92.4	-	-
ViT-SPT [32]	86.2	94.5	79.1	89.4
MBIN (Ours)	89.1	96.1	80.1	91.2

‘-’ denotes that no reported result is available.

### 4.4. Ablation Studies

To evaluate the contribution of each component in the proposed method, ablation experiments were conducted on the Occluded-DukeMTMC dataset by progressively adding or removing components. The experimental results are presented in Table 4 and Table 5.

#### 4.4.1. Effectiveness of Each Component

This study investigates the effectiveness of the MIA and VI modules. The experimental results are presented in Table 4. This study adopts PCB [13] as the baseline, which achieves a mAP of 54%, Rank-1 of 62.4%, Rank-3 of 71.4%, Rank-5 of 75.2%, and Rank-10 of 80.2%. When incorporating the MIA module alone into the baseline, the performance improves by 1.9%, 1.5%, 1.4%, 1.9%, and 1.9% in mAP, Rank-1, Rank-3, Rank-5, and Rank-10, respectively. It is shown that the MIA module enhances the high-level feature map and facilitates more effective cross-scale information extraction. Similarly, when incorporating the VI module alone into the baseline, the performance improves by 3.8%, 6.8%, 6.3%, 5.7%, and 4.5% in mAP, Rank-1, Rank-3, Rank-5, and Rank-10, respectively. It is shown that the VI module is capable of leveraging multi-view information to construct more comprehensive representations of occluded pedestrians. When both MIA and VI modules are integrated into the baseline, performance increases significantly, with mAP, Rank-1, Rank-3, Rank-5, and Rank-10 improving by 5.1%, 8.8%, 8.1%, 7.8%, and 6.5%, respectively. It is shown that the MIA and VI modules complement each other effectively. These experimental results demonstrate that introducing either the MIA module or the VI module individually into the baseline, or combining both, can enhance recognition performance.

#### 4.4.2. Effectiveness of the MKD Strategy

This study investigates the effectiveness of the MKD strategy. The experimental results are presented in Table 5. Here, $L_{\mathrm{ID}}$ represents identity loss, $L_{\mathrm{MKD}}^{V}$ represents view distillation loss, and $L_{\mathrm{MKD}}^{R}$ represents refinement distillation loss. Using only the identity loss, the method achieves mAP, Rank-1, Rank-3, Rank-5, and Rank-10 performances of 56.3%, 67.2%, 76.4%, 80.3%, and 84.3%, respectively. Combining identity loss with view distillation, mAP, Rank-1, Rank-3, Rank-5, and Rank-10 improve by 2.1%, 1.8%, 1.7%, 1.1%, and 1.4%, respectively. It is shown that the view distillation loss effectively distills the knowledge of the comprehensive pedestrian representation into both the hard and soft branches. Combining the identity loss with the refinement distillation loss, the performance improves by 2.3%, 2.3%, 1.9%, 1.4%, and 1.2% in mAP, Rank-1, Rank-3, Rank-5, and Rank-10, respectively. It is shown that the refined discriminative cues extracted from the hard and soft branches are effectively transferred to the view branch through distillation. When combining identity loss, view distillation loss, and refinement distillation loss, the performance improves by 2.8%, 4%, 3.1%, 2.7%, and 2.4% in mAP, Rank-1, Rank-3, Rank-5, and Rank-10, respectively. These experimental results demonstrate that the MKD strategy effectively exploits the complementarity between multi-branch features, promotes multi-branch collaborative optimization, and enhances the method’s recognition capabilities in occluded scenes.

### 4.5. Parameter Analysis

**Figure 3:**
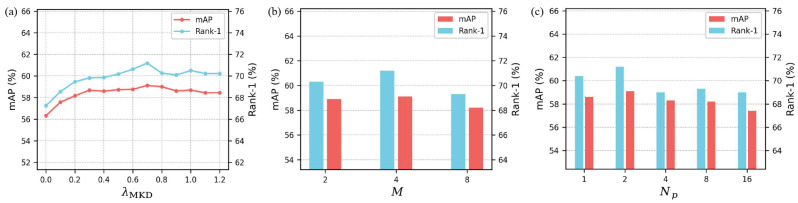
Analysis of the hyperparameters on the Occluded-DukeMTMC dataset. (**a**) Impact of hyperparameter $\lambda_{\mathrm{MKD}}$. (**b**) Impact of hyperparameter *M*. (**c**) Impact of hyperparameter $N_{p}$.

The proposed method introduces three key training hyperparameters: $\lambda_{\mathrm{MKD}}$, *M*, and $N_{p}$. To investigate their impact on performance, this study conducted experiments on the Occluded-DukeMTMC dataset. The experimental results are presented in Figure 3.

#### 4.5.1. Impact of the Hyperparameter $\lambda_{\mathrm{MKD}}$

The hyperparameter $\lambda_{\mathrm{MKD}}$ controls the strength of MKD supervision. This study evaluates $\lambda_{\mathrm{MKD}}$ in the range from 0 to 1.2, and the corresponding results are presented in Figure 3a. As $\lambda_{\mathrm{MKD}}$ increases, both the mAP and Rank-1 performance initially improve. However, further increases result in performance degradation, possibly due to an excessive reliance on knowledge transfer, which may weaken the model’s ability to learn discriminative features. Optimal performance is achieved when $\lambda_{\mathrm{MKD}}$ is set to 0.7.

#### 4.5.2. Impact of the Hyperparameter *M*

The hyperparameter *M* controls the number of images to be integrated. This study evaluates *M* at values of 2, 4, and 8, and the corresponding results are presented in Figure 3b. As *M* increases, the model benefits from multi-view information, generating richer and more discriminative representations that enhance recognition performance. However, larger values of *M* may introduce redundant or irrelevant information, thereby degrading the model’s effectiveness. Optimal performance is achieved when *M* is set to 4.

#### 4.5.3. Impact of the Hyperparameter $N_{p}$

The hyperparameter $N_{p}$ controls the number of image segments used for local feature extraction. This study sets $N_{p}$ to 1, 2, 4, 8, and 16, and the experimental results are presented in Figure 3c. As $N_{p}$ increases, each local segment contains less contextual information, making the extracted features more susceptible to occlusion and background noise. This leads to a decline in recognition performance. Optimal performance is achieved when $N_{p}$ is set to 2.

### 4.6. Qualitative Analysis

**Figure 4:**
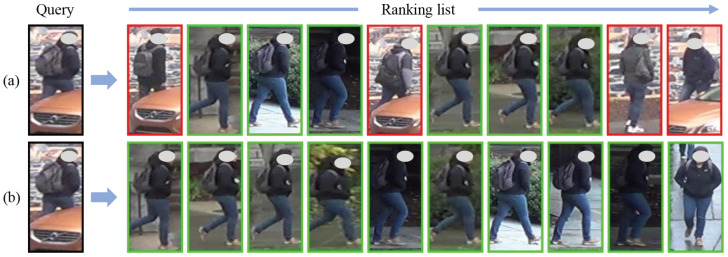
Visualization of the ranking lists on the Occluded-DukeMTMC dataset. (**a**) Results from the baseline. (**b**) Results from the MBIN.

**Figure 5:**
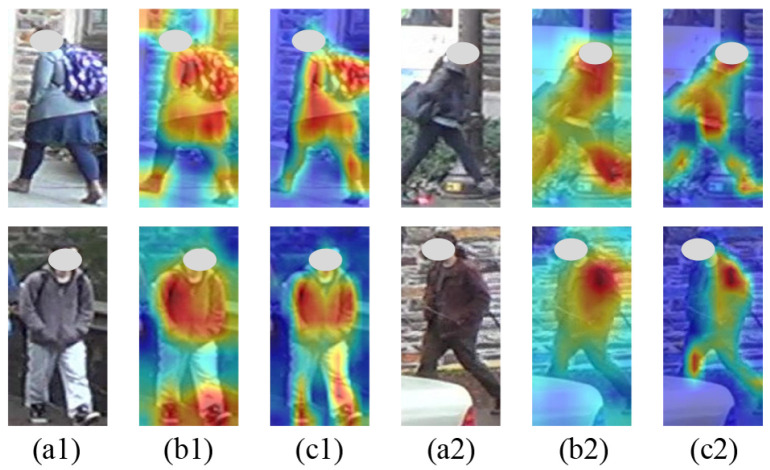
Visualization of regions of interest by the method. (**a1**,**a2**) show the original images, (**b1**,**b2**) show heatmaps generated by the baseline, and (**c1**,**c2**) show heatmaps generated by the MBIN. Colors closer to red indicate higher method attention.

To qualitatively evaluate the effectiveness of the proposed method, we conducted a visualization analysis on the Occluded-DukeMTMC dataset. The experimental results are presented in Figure 4 and Figure 5.

#### 4.6.1. Visualization of Retrieval Results

We conducted a visual analysis of the retrieval results. A target person is randomly selected from the query dataset, and ten matching pedestrian images are retrieved from the gallery dataset based on feature similarity. The experimental results are presented in Figure 4. Here, green boxes indicate retrieval results with the same identity label as the query image, while red boxes represent mismatches. When the query image is occluded, the baseline retrieves incorrect matches at Rank-1, Rank-5, Rank-9, and Rank-10. In comparison, MBIN retrieves more correct matches. These results demonstrate that the proposed method is robust and maintains high recognition performance under occlusion.

#### 4.6.2. Visualization of Heatmap

This study conducted a visual analysis of the heatmap generated using Grad-CAM [54], which highlights the regions the model attends to during person recognition. The experimental results are presented in Figure 5. In the baseline, hotspots frequently occur in background regions. It is shown that the baseline tends to rely on background information and is more susceptible to background noise during recognition. In contrast, MBIN focuses more selectively on the pedestrian’s body, effectively attending to discriminative regions. These results demonstrate that the proposed method is effective in the person re-ID task.

### 4.7. Discussion

To address the occlusion problem in re-ID, MBIN introduces a multi-branch structure that incorporates multi-head attention and a dual-classifier fusion mechanism. Mutual distillation is then applied to jointly optimize feature representations among the hard, soft, and view branches, thereby enhancing recognition performance. Some occlusion re-ID methods mainly focus on extracting features from a single branch or a multi-branch structure. For instance, compared to the single-branch method DRL-Net [21], MBIN extracts richer and more discriminative features from three branches, enhancing feature representation capabilities. As shown in Table 2, MBIN achieves higher Rank-1 and mAP performance on the occlusion dataset. Compared with the multi-branch method QPM [16], MBIN introduces the MKD strategy to achieve efficient information transfer between the three branches, thereby further improving the overall recognition performance.

This study exploits the information exchange between different branches to characterize occluded pedestrians. However, certain limitations require further attention, particularly regarding the relationship between occluded regions and recognition accuracy. Although mutual distillation improves performance, it may propagate misleading information from heavily occluded regions, potentially affecting recognition. In future work, the attention mechanism will be employed to suppress information from occluded regions.

## 5. Conclusions

This study proposes a method named Multi-Branch Interaction Network (MBIN) for occluded person re-ID. The method leverages multi-head attention to supplement the high-level feature map with multi-granularity cues, enabling better mining of pedestrian semantic information. It also employs a dual-classifier fusion mechanism to obtain a comprehensive pedestrian representation and reduce the impact of occlusions in complex environments. Additionally, the method applies mutual distillation to jointly optimize feature representations, enabling knowledge sharing among branches. Extensive experiments and analyses on person re-ID datasets demonstrate the effectiveness of the proposed method. Moving forward, the relationship between occluded regions and recognition accuracy will be further explored.

## Figures and Tables

**Table 1:** The detailed information of datasets. ’ID’ represents the number of identities.

Dataset	Train		Qurey		Gallery	
	ID	Image	ID	Image	ID	Image
Occluded-DukeMTMC	702	15,618	519	2210	1110	17,661
Occluded-REID	100	1000	100	500	100	500
P-DukeMTMC-reID	665	12,927	634	2163	634	9053
Market-1501	751	12,936	750	12,936	750	19,732
DukeMTMC-reID	702	16,522	702	2228	1110	17,661

**Table 4:** Ablation studies of MBIN on the Occluded-DukeMTMC dataset.

Method	mAP	Rank-1	Rank-3	Rank-5	Rank-10
Baseline	54.0	62.4	71.4	75.2	80.2
Baseline + MIA	55.9	63.9	72.8	77.1	82.1
Baseline + VI	57.8	69.2	77.7	80.9	84.7
Baseline + MIA + VI	59.1	71.2	79.5	83.0	86.7

**Table 5:** Effectiveness of the MKD strategy for MBIN on Occluded-DukeMTMC dataset.

Method	mAP	Rank-1	Rank-3	Rank-5	Rank-10
$L_{\mathrm{ID}}$	56.3	67.2	76.4	80.3	84.3
$L_{\mathrm{ID}}$ + $L_{\mathrm{MKD}}^{V}$	58.4	69.0	78.1	81.4	85.7
$L_{\mathrm{ID}}$ + $L_{\mathrm{MKD}}^{R}$	58.6	69.5	78.3	81.7	85.5
$L_{\mathrm{ID}}$ + $L_{\mathrm{MKD}}^{V}$ + $L_{\mathrm{MKD}}^{R}$	59.1	71.2	79.5	83.0	86.7

## Data Availability

All datasets used for training and evaluating the performance of our proposed method are publicly available and can be accessed from [24,45,46,47].
